# Temporal Dynamics of Public Emotions During the COVID-19 Pandemic at the Epicenter of the Outbreak: Sentiment Analysis of Weibo Posts From Wuhan

**DOI:** 10.2196/27078

**Published:** 2021-03-18

**Authors:** Shaobin Yu, David Eisenman, Ziqiang Han

**Affiliations:** 1 Department of Public Administration School of Political Science and Public Administration Shandong University Qingdao China; 2 Division of General Internal Medicine and Health Services Research David Geffen School of Medicine University of California Los Angeles Los Angeles, CA United States; 3 Center for Public Health and Disasters Fielding School of Public Health University of California Los Angeles Los Angeles, CA United States; 4 Center for Crisis Management Research Tsinghua University Beijing China

**Keywords:** public health emergencies, emotion, infodemiology, temporal dynamics, sentiment analysis, COVID-19

## Abstract

**Background:**

The ongoing COVID-19 pandemic has led to an increase in anxiety, depression, posttraumatic stress disorder, and psychological stress experienced by the general public in various degrees worldwide. However, effective, tailored mental health services and interventions cannot be achieved until we understand the patterns of mental health issues emerging after a public health crisis, especially in the context of the rapid transmission of COVID-19. Understanding the public's emotions and needs and their distribution attributes are therefore critical for creating appropriate public policies and eventually responding to the health crisis effectively, efficiently, and equitably.

**Objective:**

This study aims to detect the temporal patterns in emotional fluctuation, significant events during the COVID-19 pandemic that affected emotional changes and variations, and hourly variations of emotions within a single day by analyzing data from the Chinese social media platform Weibo.

**Methods:**

Based on a longitudinal dataset of 816,556 posts published by 27,912 Weibo users in Wuhan, China, from December 31, 2019, to April 31, 2020, we processed general sentiment inclination rating and the type of sentiments of Weibo posts by using pandas and SnowNLP Python libraries. We also grouped the publication times into 5 time groups to measure changes in netizens’ sentiments during different periods in a single day.

**Results:**

Overall, negative emotions such as surprise, fear, and anger were the most salient emotions detected on Weibo. These emotions were triggered by certain milestone events such as the confirmation of human-to-human transmission of COVID-19. Emotions varied within a day. Although all emotions were more prevalent in the afternoon and night, fear and anger were more dominant in the morning and afternoon, whereas depression was more salient during the night.

**Conclusions:**

Various milestone events during the COVID-19 pandemic were the primary events that ignited netizens’ emotions. In addition, Weibo users’ emotions varied within a day. Our findings provide insights into providing better-tailored mental health services and interventions.

## Introduction

Understanding the public’s emotional reactions to public health emergencies is essential in at least two ways. First, it is necessary to understand the impact of the public health crisis on the public's mental health and psychological well-being, which are the unneglected dimensions of a human being's health. The ongoing COVID-19 pandemic has led to an increase in anxiety, depression, posttraumatic stress disorder, psychological stress, and stress experienced by the general public in various degrees worldwide [1], similar to the 2003 SARS (severe acute respiratory syndrome) [2], Ebola [3], or Zika outbreaks [4]. Better tailored mental health services and interventions cannot be achieved until we understand the patterns of mental health issues that emerge after a public health crisis; this is especially true in the case of the rapidly spreading COVID-19 [5]. Thus, researchers call for multidisciplinary research for mental health as research priorities [6]. Second, the public’s collective emotions during emergencies are important in determining the emergency response to the public health crisis. Understanding the public’s emotions and needs and their distribution attributes are critical to develop appropriate public policies, as well as to nudge the public’s compliance to these policies and, eventually, respond to the health crises in an effective, efficient, and equitable manner.

There are generally two approaches to capture the public’s emotional response to an event. The traditional method involves conducting a large-scale survey, which can be both expensive and time consuming. Moreover, the self-response or retrospective bias is an inevitable criticism of the survey method. Another way is to capture the public's emotional beats on social media platforms, such as Twitter, considering the increasing use of smartphones in recent years. With the rapid development of the internet, smartphones, and social media platforms and apps, an increasing number of individuals use social media as a medium to share their opinions, social activities, and lives. Therefore, social media can be an ideal channel to capture the public’s emotions and mental health symptoms [7], especially during the COVID-19 outbreak when physical distancing policies are enforced [8-11].

Since its outbreak, COVID-19 has become an ongoing hot topic on social media. During the early phase of the outbreak, before March 15, 2020, the main topics discussed on Twitter in the English language were the origin of the virus, its sources, transmission, the impact on the economy and society, and the methods of mitigating the risks [12]. Similarly, on the Chinese social media platform Weibo, the primary concerns among users were the origin of the disease, public health control measures, disease symptoms, organizations in charge, health professionals and scientists, education sectors, economic impact, and rumor discussions [13,14]. Since the COVID-19 has lasted for such a long time and is still an ongoing pandemic, the public’s concerns and emotional responses have evolved with the pandemic's rapid change, its impacts, and countermeasure policies [15]. The declaration of the COVID-19 outbreak increased anxiety, depression, and indignation emotions among the public, whereas it decreased the public's happiness and life satisfaction [16,17]. The lockdown in Wuhan city in China and Lombardy city in Italy affected the public’s expressions on social media; moreover, a higher level of cognitive process was detected among social media users in both cities. Simultaneously, attention to group, religion, and emotions was more prevalent in Wuhan [18]. In India, a comparison between lockdown 2.0 (April 15 to May 3, 2020) and lockdown 3.0 (May 4 to May 17, 2020) revealed that the public's surprise, trust, fear, joy, and anger emotions decreased, but their anticipation, disgust, and sadness emotions increased [19]. However, policies such as physical distancing measures affected different emotions differently. A study from Spain suggested that negative emotions such as anger, fear, and disgust significantly changed, whereas sadness, joy, and uncertainty did not fluctuate much [20]. Meanwhile, positive sentiments, such as trust, anticipation, and joy, increased when discussions concerning reopening emerged on these platforms, although these emotions were mixed with negative sentiments such as fear, sadness, anger, surprise, and disgust [21].

Nevertheless, after reviewing the current sentiment analysis and emotional responses on social media, we found a lack of longitudinal observations from the declaration of the outbreak, strict enforcement of physical distancing and other constraining measures, and the reopening period. Therefore, we employed our ongoing crawled Weibo posts from 27,912 users from Wuhan to capture the emotional dynamics during a specific period of the COVID-19 pandemic, that is, from the beginning of the outbreak to the end of April 2020 (when the whole country reopened). Moreover, we divided the 24 hours of a day into several time periods to explore the emotional variations within a given day, which can be very useful for potential interventions. We used a sentiment classification algorithm that includes 6 types of emotions (ie, like, dislike, surprise, depression, anger, and fear) and analyzed the temporal dynamics of these emotions from 816,556 Weibo posts published by 27,912 users in Wuhan. In this paper, we describe (1) the temporal patterns of emotional fluctuation from the declaration of the COVID-19 outbreak in Wuhan to reopening of the city after the lockdown, (2) significant events during the COVID-19 pandemic that affected users’ emotional changes and variations, and (3) hourly variations in the netizens’ emotions expressed within a single day.

## Methods

### Data Source and Cleaning

Weibo is a social media platform like Twitter. It is the most widely used social networking platform for netizens in China to share and discuss individual opinions, events, and activities publicly. According to the 46th China Statistical Report on Internet Development report released in 2020, there were 940 million active internet users by the end of June 2020, which comprised 67% of all citizens in China [22]. The lockdown and physical distancing measures in response to the COVID-19 pandemic made the use of the internet more prevalent, and the number of Weibo active users reached its record high of 550 million in the first quarter of 2020, according to Weibo’s Q1 2020 financial report [23]. Thus, Weibo can be considered as one of the most appropriate social media channels to detect public emotions toward public events.

**Figure 1:**
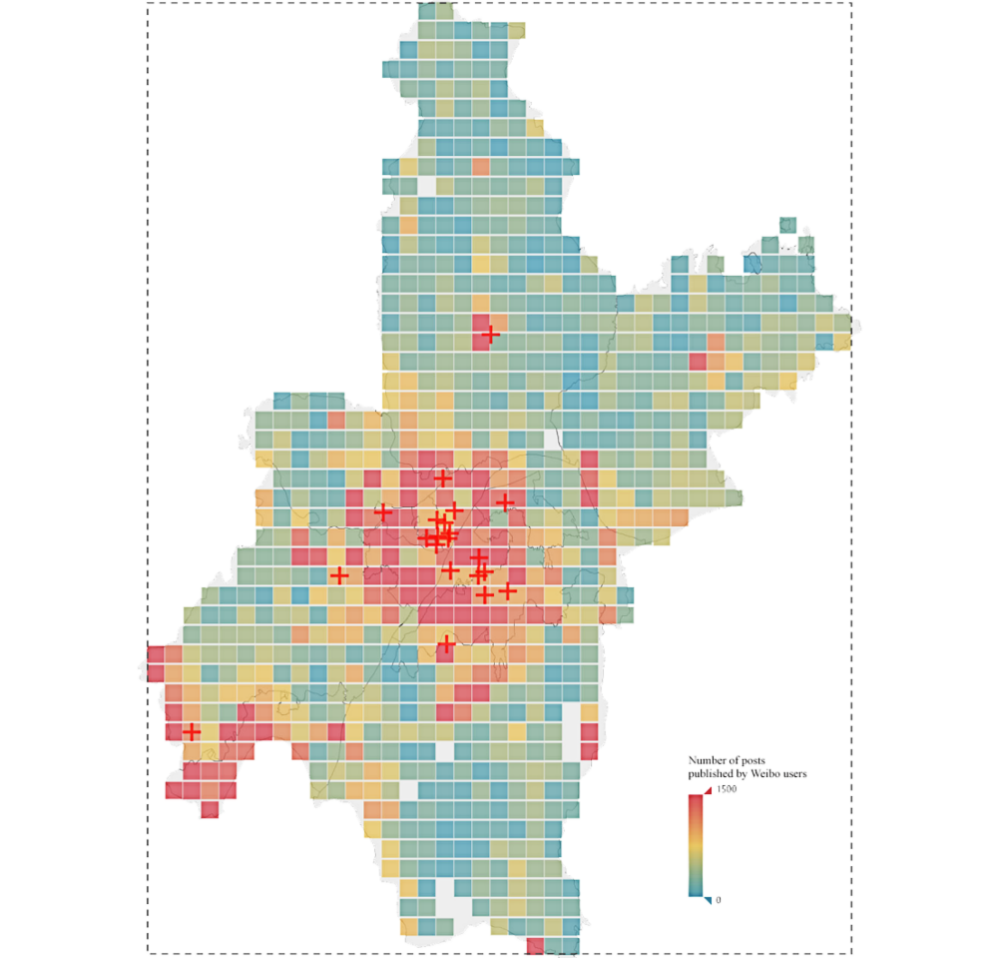
Heatmap of Weibo users’ post frequency by geolocation in Wuhan, China. Red cross symbols on the map indicates Grade-A tertiary hospital in the city.

We started to collect real-time Weibo posts as soon as a coronavirus outbreak was declared in China on December 31, 2019. For this study, we examined the dataset, including all posts posted by representative Weibo users in Wuhan from December 31, 2019, to April 31, 2020. As shown in Figure 1, we first detected the latitude and longitude of the northernmost (31.36287 N), southernmost (29.97008 N), westernmost (113.70222 E), and easternmost (115.08138 E) locations of Wuhan city. Then, we divided the quadrilateral (dotted line in Figure 1) that covered Wuhan's geographical areas into 50 segments in width and 40 segments in height. Thus, 2000 small grids were generated. Next, 50 Weibo users from each grid were randomly selected from all users located in that grid. The users’ geolocations were detected by the geotag used when they posted the message and the location of the place registered per their user profiles. Thus, 100,000 Weibo users were initially selected, from which 88,798 users were retained. The users excluded were either not located in Wuhan or duplicated because they posted multiple times using geotags from different grids or places within Wuhan's geographical area. Lastly, 27,912 users were randomly selected to participate in this study, and all of their 816,556 Weibo posts from December 31, 2019, to April 31, 2020, were collected using a web crawler. For the analysis, we collected (1) Weibo users’ profiles; (2) geolocation information of the published Weibo posts; (3) attributes of post likes, date, and time; (4) post contents; and (5) like, reply, and forward counts of the posts.

The original dataset was structured as a CSV (comma-separated values) file and processed using the pandas Python library [24]. In the data cleaning process, non-Unicode characters, empty blank spaces, and symbols were identified by regular expression patterns and then stripped. Duplicated and invalid posts were also removed. After the data cleaning process, 808,205 valid Weibo posts were selected for further analysis.

### Sentiment Analysis

Sentiment analysis is the study of emotions, opinions, appraisals, and attitudes regarding “services, products, individuals, organizations, issues, topics, events and their attributes” [25]. To explore netizens’ emotions toward COVID-19, we evaluated both general sentiment inclination and the type of sentiment, as well as their fluctuations during different periods within a day.

The general sentiment inclination rating of Weibo posts was processed by the SnowNLP library for Python [26]. SnowNLP, as one of the most popular Python libraries explicitly developed for Chinese language natural language processing, has a variety of built-in functions such as tokenization, sentiment analysis, text classification, and keyword extraction. SnowNLP also supports a self-trained dataset to improve sentiment rating accuracy for specific types of Weibo posts. Therefore, to measure sentiment ratings, a portion of the dataset was selected as the training data, and then the training result was used to reprocess the whole data and calculate the final sentiment ratings.

Specifically, we randomly selected 50,000 posts from the dataset as the training data. Thereafter, following the instructions of SnowNLP, we manually tagged each post as negative or positive sentiment and then appended the post to corresponding SnowNLP built-in corpus files that were named as neg.txt and pos.txt, respectively. The updated sentiment rating corpus files were then fed into SnowNLP for training. The result was the newly generated sentiment pattern file named sentiment.marshal in the SnowNLP library folder. Based on the training result, all of the 808,205 posts were evaluated, and sentiment ratings were remeasured. The result was a score ranging from 1 to 10 for each post, denoting the most negative to the most positive sentiments. We then divided each sentiment rating value by 10, and the final sentiment ratings ranged from 0.1 to 1.

### Sentiment Type Analysis

To properly align each post's sentiment type, this study used the DUTIR Emotion Ontology set, developed and maintained by Prof Lin Hongfei and his team at Dalian University of Technology Institute of Information Retrieval (DUTIR), as the emotion lexicon resource [27]. Their study compared the tokenized words with the DUTIR Emotion Ontology set and generated a list of words with their corresponding emotional categories for each Weibo post. After that, the word in the emotional category with the most frequent occurrences was treated as the post's baseline sentiment type. Then, each Weibo post was tagged with a sentiment type—depression, like, angry, dislike, surprise, or fear.

Both clinical and self-reported studies indicate that people’s moods, such as depression [28,29] or rumination [30], vary within a day. To measure netizens’ sentiment changes during different periods in a day, we grouped the hours into 5 time groups: early morning (04:00 to 08:00), morning (08:00 to 12:00), afternoon (12:00 to 17:00), evening (17:00 to 20:00), night (20:00 to 00:00), and late night (00:00 to 04:00). We grouped all of the general sentiment inclinations by day and time using the pandas library’s *groupby* method and counted each group’s frequencies. Based on the result, we calculated the ratio of each sentiment type in the specific period, that is, the percentage of occurrences of each sentiment type in the specific time group.

## Results

### Geolocation Distribution of Weibo Users and Post Frequencies

First, we plotted all of the Weibo users on the map of Wuhan according to their geolocation information. We drew a heatmap overlaid on the city map representing the total number of Weibo posts by users during the whole period. The final output shows the frequency of posts in different locations of Wuhan. Additionally, we plotted the representative Grade-A tertiary hospitals in Wuhan on the map to better understand the relationship between Weibo users’ post patterns and their proximity to neighboring hospitals. We used locations of the major hospitals as a proxy of the places where newly confirmed COVID-19 cases occurred, and the distances can reflect the proximity to these places and citizens’ posting behaviors. The map demonstrates a clear correlation pattern between the number of Weibo posts published by users and their physical distance to major hospitals.

### Temporal Evolution of Sentiment Types

**Figure 2:**
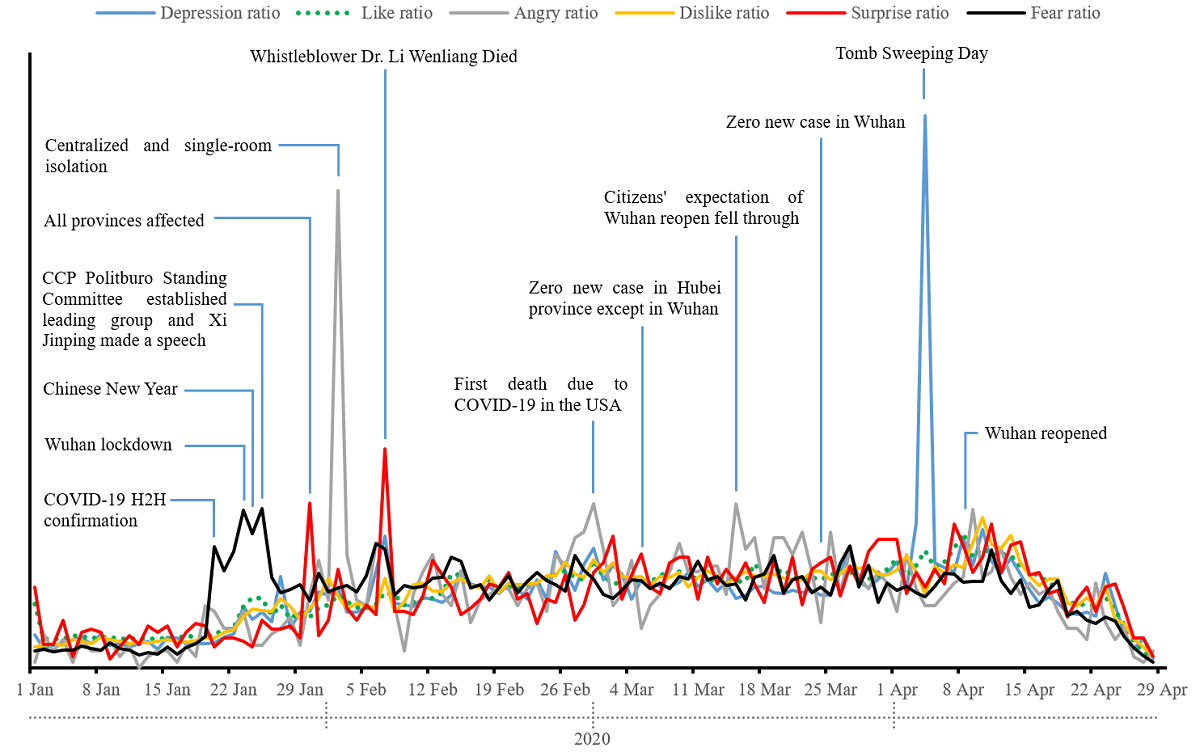
Line chart of the temporal evolution of sentiment types based on Weibo posts from Wuhan, China.

The temporal evolution of different sentiment types is illustrated in Figure 2. The higher the value, the stronger the type of sentiment. Overall, fear, anger, and surprise were the most dominant emotions, whereas like, dislike and depression were the less salient emotions. Similar variations and dynamics were observed for the negative emotions during the analysis. The emotional responses can be broadly differentiated into three periods. First, there was a sharp increase in all sentiments after the confirmation of human-to-human transmission of COVID-19 on January 20, 2020; thereafter, this increasing trend remain steady until February 7, 2020, when the young whistleblower Dr Li Wenliang passed away. Second, the number and variations of posts on Weibo were considerably higher shortly after the confirmation of human-to-human transmission of the virus compared with posts published later. Moreover, a subtle and smooth pattern was maintained across all emotions, with fewer variations noted until Wuhan reopened after April 8, 2020. The third period was after the lockdown was lifted (ie, reopening); a significant decrease of posts and all emotions was observed during this period.

The result also demonstrated that Wuhan residents' emotions were significantly affected by milestone events. Fear was the dominant emotion in the first week; it reached the first peak on January 20, 2020, when human-to-human transmission of COVID-19 was confirmed and then increased to a higher peak on January 23, 2020, with the declaration of the city entering a lockdown. Another peak for fear was recorded one day later when President Xi deployed the emergency response on the first day of the Chinese New Year. With regard to the other emotions, the first peak for depression was reached shortly after the first day of the Chinese New Year. The first peak of surprise was recorded on January 30, 2020, when Tibet, the last province of China to report a COVID-19 outbreak, reported new COVID-19 cases, indicating that all the provinces of Mainland China had reported new positive cases of COVID-19 that day. The first and the highest peak for anger appeared on February 2, 2020, when all infected or suspected patients, those with symptoms similar to COVID-19, or close contacts of infected patients were required to be collectively isolated. The emotions declined on February 5, 2020, when President Xi gave another concrete national response direction. Thereafter, all negative emotions (ie, fear, anger, surprise, and depression) increased and reached a new peak on February 7, 2020, when the public recognized the young doctor Li Wenliang passed away. After these events, all the emotions maintained a steady trend at a low level except for two peaks observed for anger. One peak was observed on February 29, 2020, when the first COVID-19–related death occurred in the United States, and the local officials of Wuhan declared that the city had adequate supplies for food and living goods that can last at least 1 month. The other peak was observed on March 15, 2020, when the public’s expectation of the city reopening did not come true. The news about zero new confirmed COVID-19 cases, either in Wuhan or in other places within Hubei province except for Wuhan, had little influence on the residents' emotions. The depression emotion had reached the highest peak and was the dominant emotion around the Tomb Sweeping Day (April 4, 2020), the traditional memorial festival in the Chinese culture for the reverence of ancestors and the passed ones.

### Intraday Distribution of Sentiment Types

**Figure 3:**
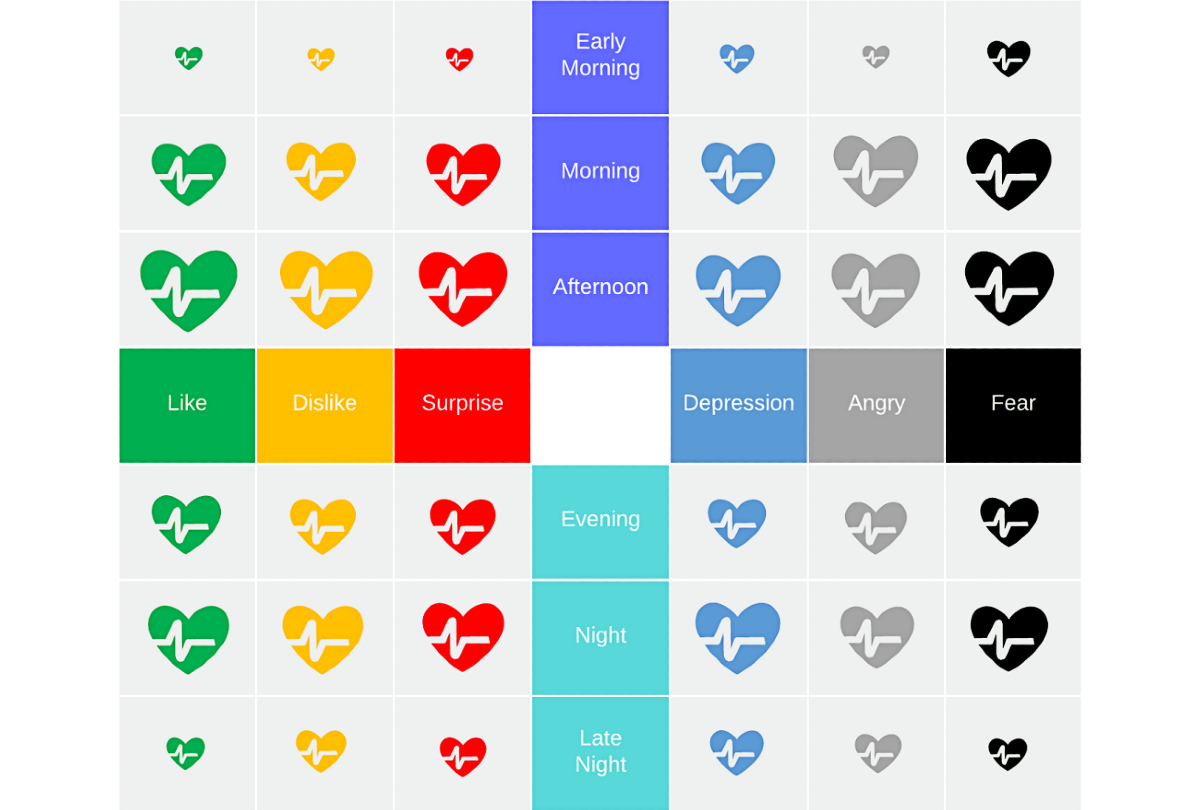
Distribution of different types of sentiments across different time periods used for analysis. The bigger the heart symbol, the stronger the corresponding emotion was during the given time period.

The variations of emotions within a day are illustrated in Figure 3. We computed the mean intensity of each sentiment type during different periods (ie, early morning, morning, afternoon, evening, night, and late night) and presented them in a matrix diagram. Afternoon (12:00-17:00) and night (20:00-00:00) were the primary periods when people use social media to express their feelings during the COVID-19 epidemic, followed by morning (08:00-12:00), evening (17:00-20:00), late night (00:00-04:00), and early morning (04:00-08:00). In the morning period, fear and anger were the two most dominant emotions. In the afternoon period, like and dislike emotions were the highest (or most dominant), although most emotions expressed on social media were noted during this period. During the night period, depression was the most dominant emotion.

### Temporal Evolution of Sentiment Types During Different Time Periods

**Figure 4:**
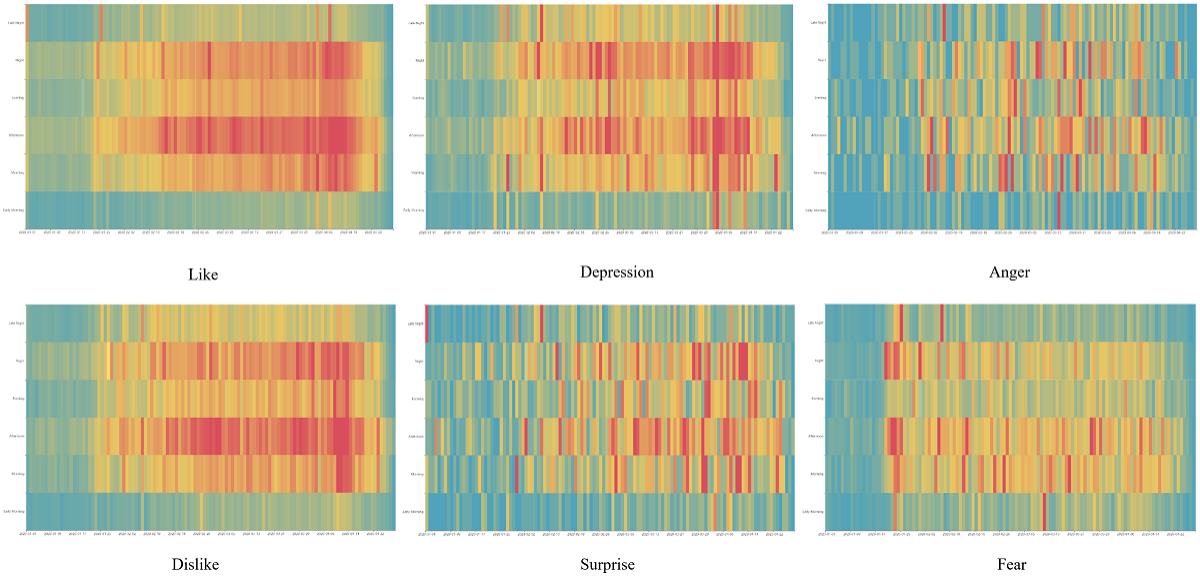
Temporal changes in sentiment type by days (horizontal) and hours (vertical).

To better understand the temporal distribution of emotions, we used heatmaps to demonstrate the distribution of each emotion by days and hours within a given day (Figure 4). A stronger emotion is presented in a darker red color. The relatively gentle emotions such as like and dislike were consistent during the lockdown period, especially in the afternoon and night periods. The relatively stronger emotions such as anger and fear appeared periodically, likely triggered by different major events. The emotion depression was also prevalent over time, especially after the young whistleblower doctor passed away and on the Tomb Sweeping day, commemorated in honor of those who have passed away.

## Discussion

### Principal Findings

In this study, we followed the emotional changes of 27,912 social media (Weibo) users from Wuhan, China, from December 31, 2019, to April 31, 2020, covering all the stages before the COVID-19 outbreak, declaration of the outbreak, the lockdown, and the subsequent reopening of the city. Specifically, we analyzed the temporary dynamics of users’ sentiments, including like, dislike, surprise, depression, anger, and fear, expressed through the Weibo platform and displayed these emotions across daily and hourly periods. There are at least three noteworthy findings from this study.

First, negative emotions such as surprise, fear, and anger were the dominant emotions on social media since the confirmation of the human-to-human transmission of the virus. In the very early stage of the outbreak (ie, first 2 weeks), fear was the dominant emotional response, when the virus was first detected, and human knowledge about this virus and its impact was minimal. Anger is another dominant emotion that is easily influenced by milestone events. An earlier study has demonstrated that anger was more influential on Weibo than emotions like joy or sadness [31], and a similar trend was observed during the COVID-19 outbreak. The other emotions—like, dislike, and depression—mildly fluctuated in the first half-year of 2020, and all emotions decreased significantly when the city was reopened, but there was a short-term increase in these emotions several days immediately after the reopening of the city post the lockdown. Our finding is similar to a previous study that used a search index of psychological keywords from residents in Wuhan; the search of keywords specific to fear and psychological counseling increased significantly in the first month after the lockdown, compared with the same period in 2019, whereas this was not true for keywords specific to depression and insomnia [32].

Another novel finding of our study is that we detected hourly variations in the emotions within a day, which researchers have rarely paid attention to before. We found that netizens from Wuhan expressed more emotions in the afternoons (12:00-17:00) and nights (20:00-00:00). The emotions anger and fear were more prevalent in the mornings and afternoons, whereas the emotion depression was relatively more prevalent during nighttime. This observation is similar to those of prior studies that have investigated the diurnal variations of mental health–related behaviors. Google search data from Finland shows that depression-related query volumes started to increase in the late evenings and reached a peak around midnight [33], although Canadian police records indicate that the call volumes for mental health service generally peaked in the mid-afternoon [34]. Our study using social media data reconfirms the hourly variations of mental health symptoms, and this finding has potential contribution to the implementation of mental health intervention strategies in the future.

Moreover, the pandemic's evolution and the response measures significantly affected people's emotions, especially anger, in the whole process. It was observed that the general quarantine measures did not have a negative psychological effect on the affected individuals in case of the less-deadly disease like H1N1 [35], but this was not true for the more-deadly disease such as SARS [36]. However, COVID-19—as a collective stressor—reduced the general public's mental health and psychological well-being worldwide [37-40]. Different concerns during different stages of the pandemic evolution are associated with different emotions on social media. For example, fear could be related to the shortage of medical and test supplies; anger could be associated with xenophobia, at first, and with the stay-at-home orders, later; sadness could cooccur with the topics of losing friends or family members; and joy could be associated with words of gratitude and good health [15]. For residents of Wuhan, fear primarily occurred during the first 2 weeks of the outbreak, and the confirmation of human-to-human transmission of the disease, the city's lockdown, and the declaration of the national response by President Xi; moreover, the passing away of the whistleblower Dr Li caused the fear emotion to peak. With the occurrence of positive COVID-19 cases in all provinces of China, Dr Li's death led to a peak of the surprise emotion. The anger emotion first reached an outstandingly high level when a decision was made to quarantine and isolate all infected patients and their close contacts in Wuhan. Other peaks during the process were observed on the day the first confirmed death due to COVID-19 was reported in the USA, which was also the day when the Wuhan city government declared that the city had a full capacity of logistics and supplies, and the day when the public's expectation of city reopening failed. Moreover, the depression emotion exploded on Memorial Day and reached another peak when Dr Li passed away. These findings suggest that public health emergency response strategies should be tailored to the dynamics and evolution of the disease and the public's response [41].

Furthermore, our sampling and data collection can provide methodology insights for studies using social media data. Unlike the studies using keyword search methods, those based on the social media platform’s feedback through application programming interfaces [42], those exploring only a small number of social media users, or those covering only a short period [16,18,43], we used a relatively sophisticated sampling method and developed a “longitudinal” dataset of social media posts published by a large sample of respondents (Weibo users). Our analysis and visualization methods can also prove to be a valuable contribution to future studies investigating and presenting netizens’ opinions and behavioral changes.

### Limitations

Nevertheless, there are at least two limitations of our study. The first is that we did not link Weibo users' profiles with their posts and emotional dynamics. If we can detect the demographic variations of these emotional changes, it may be better to detect the psychological needs of the residents during a public health emergency response, especially in the context of the lockdown of a city. However, there could be ethical and privacy concerns of including the detailed profile information of social media users [44,45], particularly considering those who are unwilling to reveal their mental health status. Hence, we anonymized all users’ information in our analysis. Second, we did not include the users’ behavioral changes, including both protective behaviors and those that have a negative impact (eg, alcohol consumption) in this analysis. We also did not cover posttraumatic stress disorder and anxiety emotions, which can also be prevalent and vital during the COVID-19 pandemic [46,47].

### Conclusions

In this study, we investigated temporal emotions (like, dislike, surprise, fear, depression, and anger) and their dynamics from nearly 30,000 Weibo users in Wuhan from December 31, 2019, to April 31, 2020, covering different periods, ranging from before the COVID-19 outbreak, the resulting city-wide lockdown, and the subsequent reopening of the city. Milestone events were the primary triggers that ignited various emotions during this period. Negative emotions such as surprise, fear, and anger were the most salient emotions on the Chinese social media platform Weibo. The emotions also varied within a given day. All emotions analyzed were more prevalent in the afternoon and night, but fear and anger were more likely to appear in the morning and afternoon, and depression was more salient during the night. Our finding can potentially contribute to the implementation of mental health intervention strategies in the future. In addition, our analysis and visualization methods can also prove to be a valuable contribution to future studies investigating and concerning netizens’ opinions and behavioral changes.

## Abbreviations

DUTIR: Dalian University of Technology, Institute of Information Retrieval
SARS: severe acute respiratory syndrome
